# Three-Dimensional X-ray Observation of Atmospheric Biological Samples by Linear-Array Scanning-Electron Generation X-ray Microscope System

**DOI:** 10.1371/journal.pone.0021516

**Published:** 2011-06-23

**Authors:** Toshihiko Ogura

**Affiliations:** Biomedical Research Institute, National Institute of Advanced Industrial Science and Technology (AIST), Umezono, Tsukuba, Ibaraki, Japan; The George Washington University, United States of America

## Abstract

Recently, we developed a soft X-ray microscope called the scanning-electron generation X-ray microscope (SGXM), which consists of a simple X-ray detection system that detects X-rays emitted from the interaction between a scanning electron beam (EB) and the thin film of the sample mount. We present herein a three-dimensional (3D) X-ray detection system that is based on the SGXM technology and designed for studying atmospheric biological samples. This 3D X-ray detection system contains a linear X-ray photodiode (PD) array. The specimens are placed under a CuZn-coated Si_3_N_4_ thin film, which is attached to an atmospheric sample holder. Multiple tilt X-ray images of the samples are detected simultaneously by the linear array of X-ray PDs, and the 3D structure is calculated by a new 3D reconstruction method that uses a simulated-annealing algorithm. The resulting 3D models clearly reveal the inner structure of the bacterium. In addition, the proposed method can easily be used for diverse samples in a broad range of scientific fields.

## Introduction

Imaging with nanometre-scale resolution is indispensable for many scientific fields such as biology, physics, chemistry, materials science and nanotechnology [1]–[5], and soft-X-ray microscopy is an important technique for producing such high-resolution images of unstained samples that are under atmospheric pressure or in water [6]–[9]. When combined with a diffractive zone plate and a synchrotron light source, the spatial resolution of soft-X-ray microscopy is approximately 10 nm [10], [11], which is much higher than the resolution available with an optical microscope. Moreover, X-ray microscopes that exploit the computer-tomography (CT) method can image the three-dimensional (3D) structure of diverse samples [12]–[16]. However, these methods require large and complex synchrotron light sources. Furthermore, 3D reconstructions using these systems require the acquisition of many images with the sample rotated and/or tilted between each image, and acquiring these numerous images can lead to sample damage. Moreover, to acquire an image at each viewing angle, the beam focus and the sample position must be adjusted between each image, which leads to long data-acquisition times.

Recently, we have developed a soft-X-ray microscope called the scanning-electron generation X-ray microscope (SGXM) [17]–[19]. This microscope consists of a simple system for detecting the soft X-rays generated by the interaction between a scanning electron beam of a scanning-electron microscope (SEM) and a target material layer on thin film. Therefore, X-ray imaging of various samples is relatively simple with SGXM. Furthermore, the spatial resolution of the SGXM incorporated into a field-emission SEM is 11 nm [19] and its theoretical resolution is less than 5 nm [18].

In this paper, we present a 3D reconstruction system based on the SGXM technology for studying atmospheric biological samples. The X-ray detection system consists of a linear X-ray photodiode (PD) array; hence, multiple sample tilt images are acquired simultaneously by PDs during a single electron-beam (EB) scan. Moreover, we have developed a highly accurate 3D reconstruction method that uses a simulated-annealing (SA) algorithm.

Currently available methods for 3D volume reconstruction include the filtered-back projection method, the algebraic reconstruction technique and the simultaneous iterative reconstruction technique [20], [21]. However, these methods require numerous projections. Some two-dimensional (2D) CT methods have been developed that use the SA algorithm and enable highly accurate reconstructions from a reduced number of projections [22]–[24]. Furthermore, we have developed a 3D reconstruction method that uses an SA algorithm and have applied it to imaging proteins with a transmission electron microscope [25].

We, thus, propose herein a 3D reconstruction method that uses an SA algorithm combined with images captured by a linear array of X-ray PDs. The atmospheric sample holder, the linear-array X-ray PD system and the SA 3D reconstruction algorithm constitute a new technology that is expected to contribute in a broad range of scientific fields for analysing the 3D structure of various atmospheric samples.

## Results

**Figure 1:**
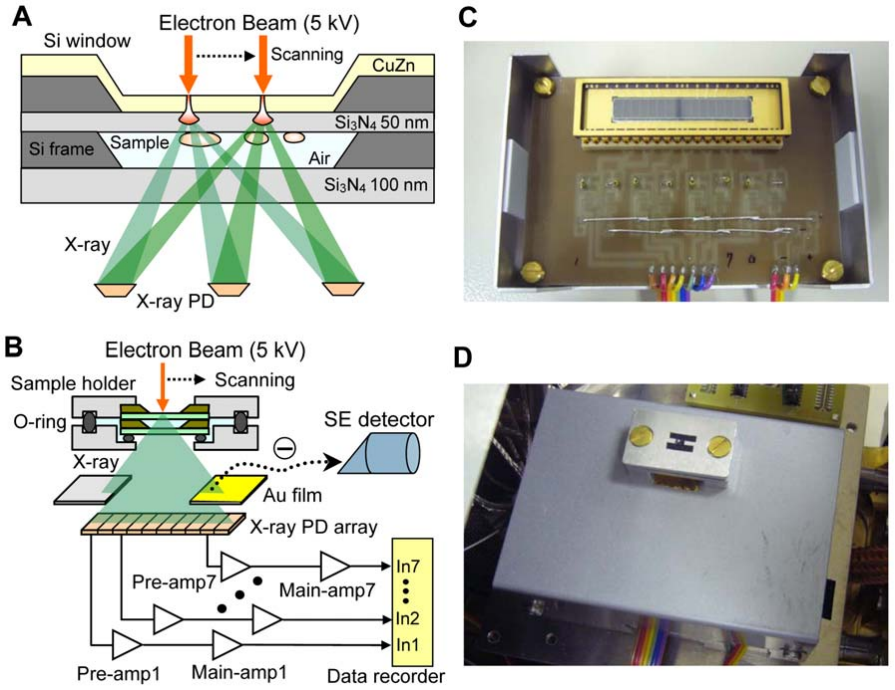
Experimental setup and new 3D-SGXM system. (A) Schematic of the atmospheric sample holder. Bacteria samples are positioned under the CuZn-coated Si_3_N_4_ film; these samples are maintained at atmospheric pressure. A scanning 5-kV EB irradiates the upper side of the CuZn-coated Si_3_N_4_ film. The linear X-ray PD array under the sample detects the X-rays transmitted through the sample. (B) Schematic of X-ray-detection apparatus. The X-ray images are acquired by both the SE detector (which detects the photoelectric conversion electrons) and the linear X-ray PD array. The X-ray image acquired from the SE detector was obtained from the photoelectrons generated at the Au-coated film, which is positioned at the side of the PD stage. A linear X-ray PD array consisting of seven PDs was fixed under the sample, and its output was recorded using a data recorder (EZ7510, NF Co., Japan) after amplification by PD preamplifiers (gain 100× MA/V) and main amplifiers (gain 100×). (C) Photograph of the linear X-ray PD array system. The linear X-ray PD array is visible in the upper centre on a circuit board. In this system, the X-ray signals are obtained by seven X-ray PDs (XPD 1–7) from among the 16 elements, and the output from each X-ray PD is amplified by a preamplifier, which is located under the circuit board. (D) Photograph of atmospheric sample holder set upon the X-ray detection system. The linear X-ray PD array system is covered by an Al box, but its position is marked by the hole that is visible beneath the sample holder. An atmospheric sample holder was mounted on the Al stage. The Au-coated photoelectron conversion film is fixed in the gap between the sampler holder and the X-ray detection box.

A schematic of the atmospheric 3D-SGXM system is shown in Figure 1. The unstained sample is positioned under a brass (CuZn)-coated Si_3_N_4_ film and sealed by a 100-nm Si_3_N_4_ film (Figure 1A), and the ensemble is attached to an atmospheric sample holder (Figure 1B) in which the sample space is maintained at atmospheric pressure. The sample holder is then mounted on the stage of the linear X-ray PD array system combined with a photoelectric conversion film (Figure 1B–D).

The CuZn-coated Si_3_N_4_ film is scanned by an EB accelerated at a voltage of 5 kV; hence, the impinging electrons are almost absorbed on the CuZn-coated Si_3_N_4_ film (Figure S1). Consequently, electron damage to the sample is strongly suppressed. In addition, the interaction of the scanning EB with the CuZn thin layer emits approximately 1-keV X-rays (Figure S1) that irradiate the sample.

The X-rays transmitted through the sample are detected by the linear PD array and photoelectron conversion systems (Figure 1B). To form X-ray images, photoelectrons from the Au-coated film are detected by a secondary-electron (SE) detector in the SEM, which is typically supplied with conventional SEMs. This process constitutes the photoelectron conversion system [19]. As a result, X-ray images can easily be observed because the same SEM position setting and focus can be used.

To form X-ray images from the linear PD array, the X-ray signals are read from the seven PDs that are used from among the 16 elements at one PD interval (Figure S2). The seven PD outputs are amplified by preamplifiers and main amplifiers and then recorded with a data recorder (Figure 1B). Because of their linear arrangement, each PD views the sample from a different angle and hence collects a different tilt image. The angles between the EB-irradiated spot in the Si_3_N_4_ film and the PD elements range from −40.2° to 45.0° (Figure S2). Finally, the X-ray images are reconstructed from the X-ray signals combined with the EB scan signal and the PD angle corrections.

**Figure 2 pone-0021516-g002:**
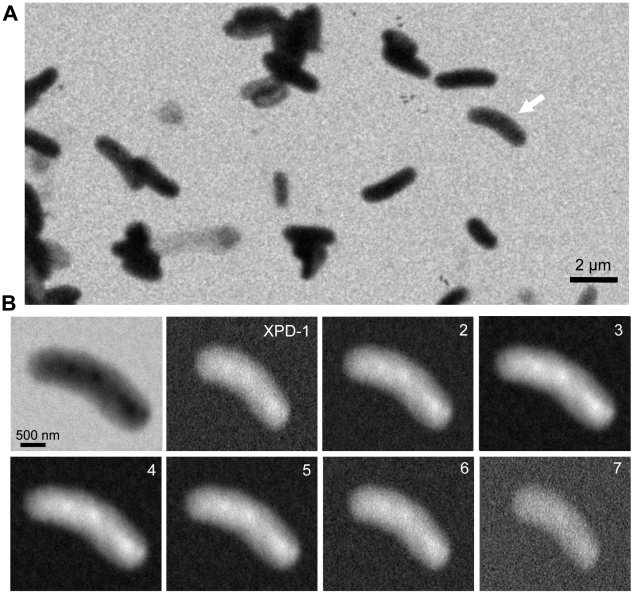
X-ray image of atmospheric unstained bacteria by SGXM system. (A) X-ray image of the atmospheric *Rhodobacter capsulatus* acquired by the photoconversion system using an original SE detector. The image was acquired at 4000× magnification at a 5-kV EB acceleration voltage. The image was filtered by a 2D Gaussian filter (size 15×15 pixels; σ = 1). (B) High-resolution X-ray images of the bacterium indicated by white arrow in panel (A). The top left image shows an X-ray image of a bacterium acquired with the SE detector and taken at 30,000× magnification. The X-ray images from XPD 1–7 were acquired by the linear PD array. Each X-ray image was corrected for the view angle and X-ray absorbance. In these images, XPD-1 is the tilt image at −40.2°, XPD-4 is placed approximately under the sample (4.4°) and XPD-7 is at 45°.

With the 3D-SGXM system, we first observed unstained atmospheric bacteria of *Rhodobacter capsulatus* [26], [27]. Figure 2A shows an X-ray image of the bacteria positioned under the CuZn-coated Si_3_N_4_ film from the SE detector, acquired at 4000× magnification at a 5-kV EB. The unstained bacteria create a black contrast and have an elongated shape, which is 2 to 5 µm long and approximately 1 µm wide. The bacterium indicated by the white arrow in Figure 2A was scanned at 30,000× magnification and is shown in the top-left panel of Figure 2B. The inner structure of this bacterium is clearly visible at this magnification.

Figure 2B shows the simultaneously acquired tilt images of the same bacterium obtained from the linear PD elements and corrected for angle and X-ray absorbance. In these images, X-ray PD-1 (XPD-1) views the sample at *θ*_1_ = −40.2°; XPD-4 is placed approximately under the sample (*θ*_4_ = 4.4°) and XPD-7 views the sample at *θ*_7_ = 45° (Figure S2). To correct the tilt angle, the horizontal axes of the detected images are reduced by a factor cos(*θ_x_*). Furthermore, in the 3D model, X-ray images are converted to X-ray absorbance after angular correction (see Materials and Methods).

**Figure 3 pone-0021516-g003:**
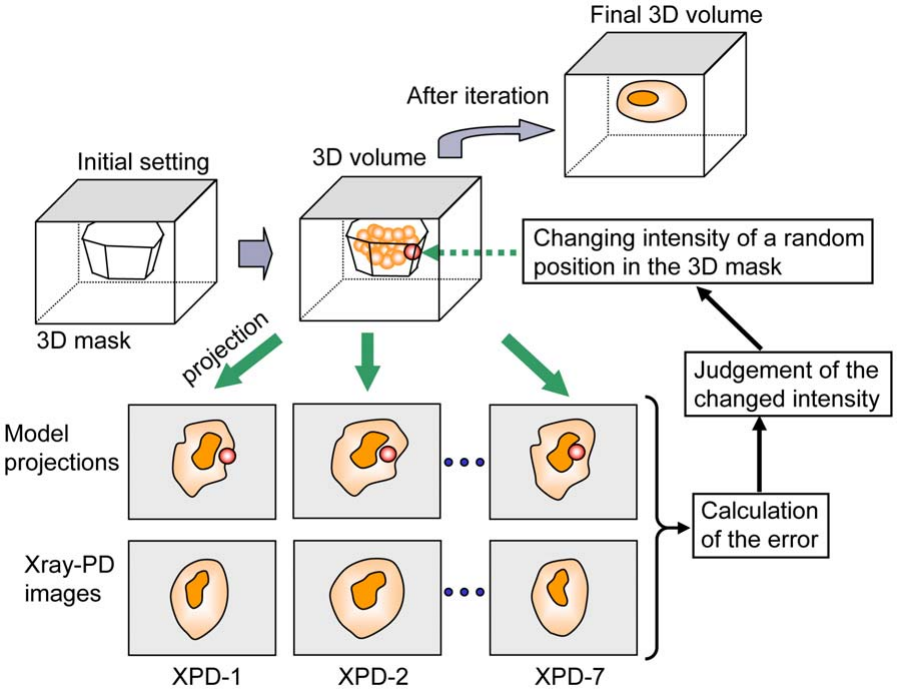
Outline of new 3D reconstruction method that uses SA algorithm. First, a 3D mask is calculated from the X-ray images of the sample. Next, a position in the 3D mask is selected at random and its voxel values within the kernel-boundary area are altered at random. The thus-modified 3D volume is reprojected onto the seven angular directions to the X-ray detectors. To evaluate the modified values, the residual sum of the squares between all projections and its corresponding X-ray images is calculated and used by the SA algorithm to determine whether to accept the new 3D volume. This procedure is iterated 100,000 times, and when finished, the temperature, the kernel size and its value-shifting SD decrease exponentially. These processes are iterated for a prefixed cycles.

**Figure 4:**
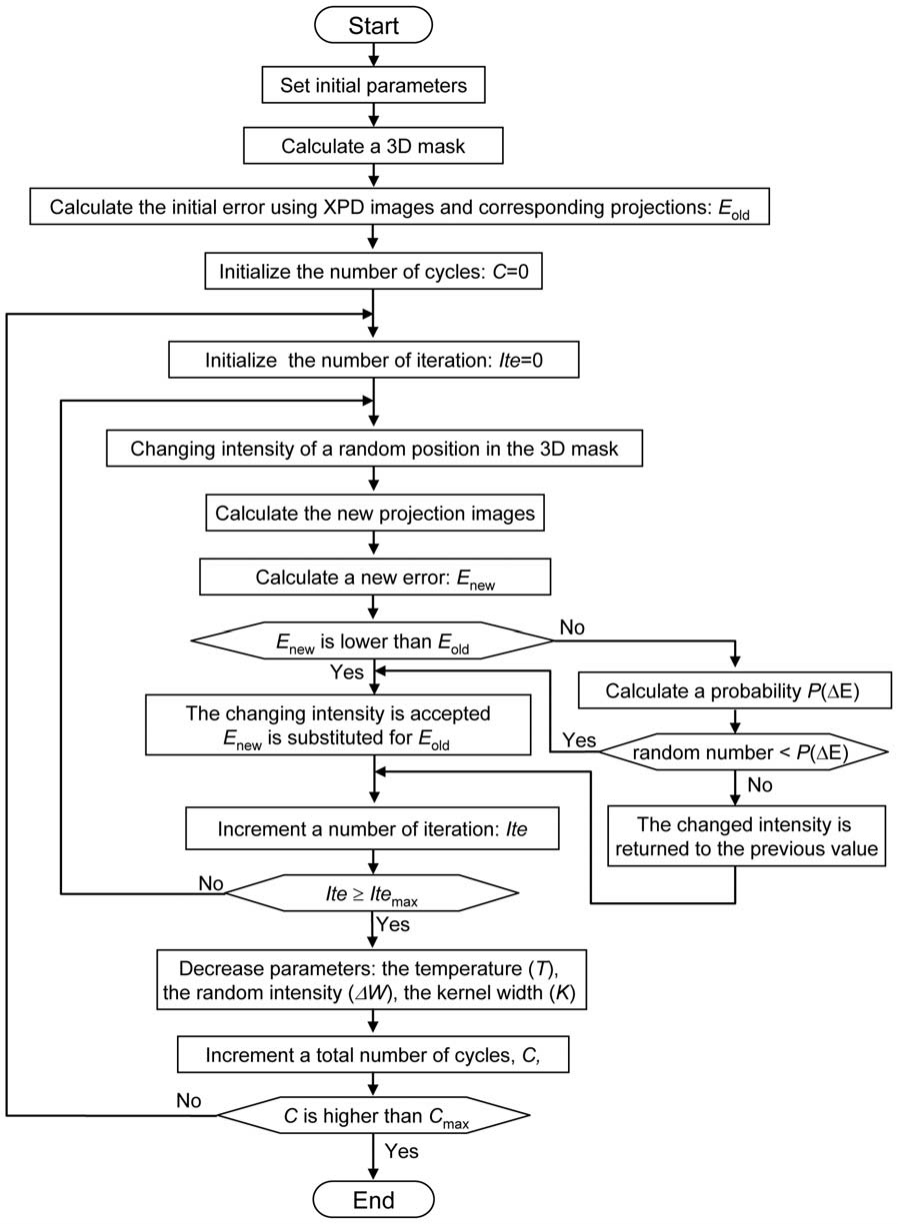
Flowchart of newly developed 3D reconstruction method employing SA algorithm. First, the 3D mask of the sample is calculated from the tilt X-ray images acquired by the linear X-ray PD array. Next, we select a random position in the 3D mask, change its voxel values in a random shift, calculate the new error from the new 3D volume and feed this new error to the SA algorithm to determine whether to accept the modification. The error value used to determine whether to accept the modification is the residual sum of the squares of the projections from the new 3D volume and its corresponding X-ray images. This process is iterated for a predetermined number of times (100,000 times for the work reported herein). The temperature and kernel of changing voxels are thereby gradually reduced. These steps are iterated for a predetermined number of cycles (200 cycles for the work reported herein).

To construct a high-accuracy 3D model from seven X-ray images, we developed a new 3D reconstruction method that uses the SA algorithm (Figures 3 and 4). This algorithm enables 3D reconstruction from a small number of projections, even if its projections are restricted tilt angles. A schematic of the proposed 3D reconstruction method is shown in Figure 3 and its flow diagram is given in Figure 4. Initially, a 3D mask is calculated by back projecting the 2D masks obtained from the X-ray images (Figure S3). The 3D voxel values and the seven projections are initially set to 0. Next, a position in the 3D mask is randomly selected and its voxel values within the kernel-boundary area are randomly shifted. This new 3D volume is reprojected onto the seven angles of the corresponding PDs. To evaluate the modified voxel values, the residual sum of the squares between all reprojections and its corresponding X-ray images is calculated and used by the SA algorithm to determine whether to accept the new 3D volume (Figure 4). This procedure is iterated 100,000 times. When finished, the temperature, the kernel size and is the standard deviation (SD) decrease exponentially. The kernel area begins with an SD of 15σ voxels, which is finally reduced to 2σ voxels. The entire cycle is repeated 200 times.

**Figure 5:**
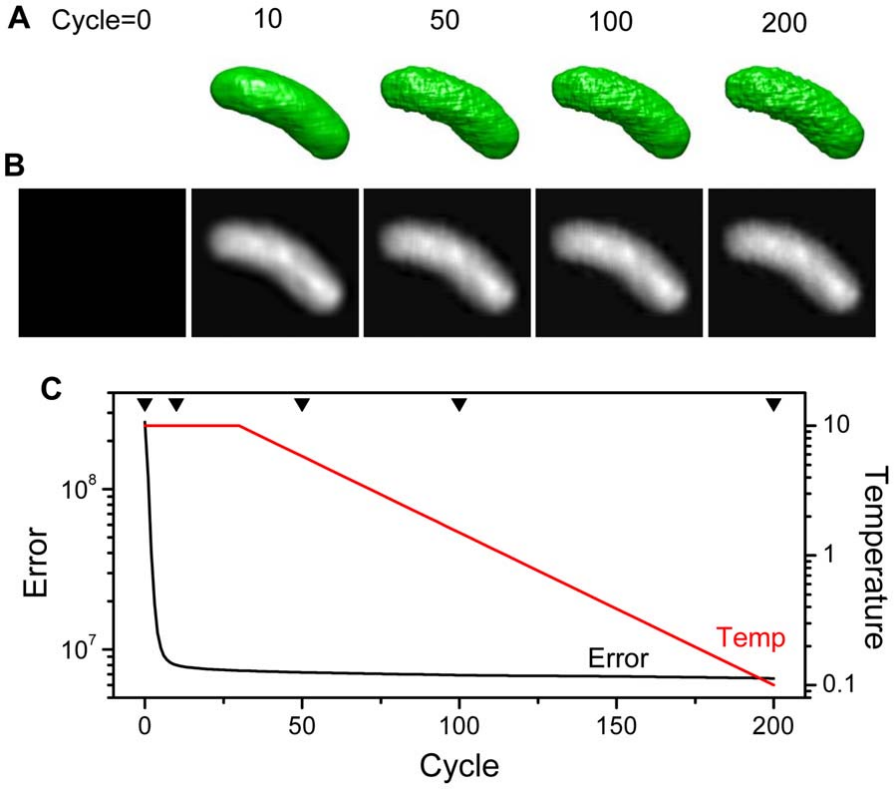
Evolution in 3D reconstruction and projection of XPD-4 at annealing cycles. (A) Evolution of 3D structure as a function of the number of annealing cycles specified above each 3D image. Initially, the 3D volume is blank. Starting from the blank volume, the 3D volume rapidly emerges in just 10 cycles. Beyond 10 cycles, the 3D volume is gradually optimized and the fine structure appears. (B) Evolutions of the projection image corresponding to XPD-4 angle (4.4°) after the specified number of annealing cycles. (C) Error and temperature as a function of annealing cycles. The temperature remains constant at 10 for the first 30 cycles. After 30 cycles, the temperature decreases exponentially. However, within 10 cycles, the error is rapidly reduced. After 10 cycles, the error decreases gradually.

We applied our 3D reconstruction method to the tilt X-ray images of a bacterium sample (Figure 2B). The modifications in the 3D volume and the XPD-4 model projections between various annealing cycles are shown in Figure 5A and B, respectively. Starting from the blank volume and its projected image (left-most panels in Figure 5A and B), the 3D volume and the projection rapidly emerge after just 10 cycles, at which point the projection already approximates the X-ray images. After 200 cycles, the projection is very similar to the corresponding X-ray image. Figure 5C shows the annealing temperature and the error between the projections and the X-ray images as functions of the cycle number. Between 0 and 10 cycles, the error decreases drastically and then it decreases gradually with the cycle number.

**Figure 6 pone-0021516-g006:**
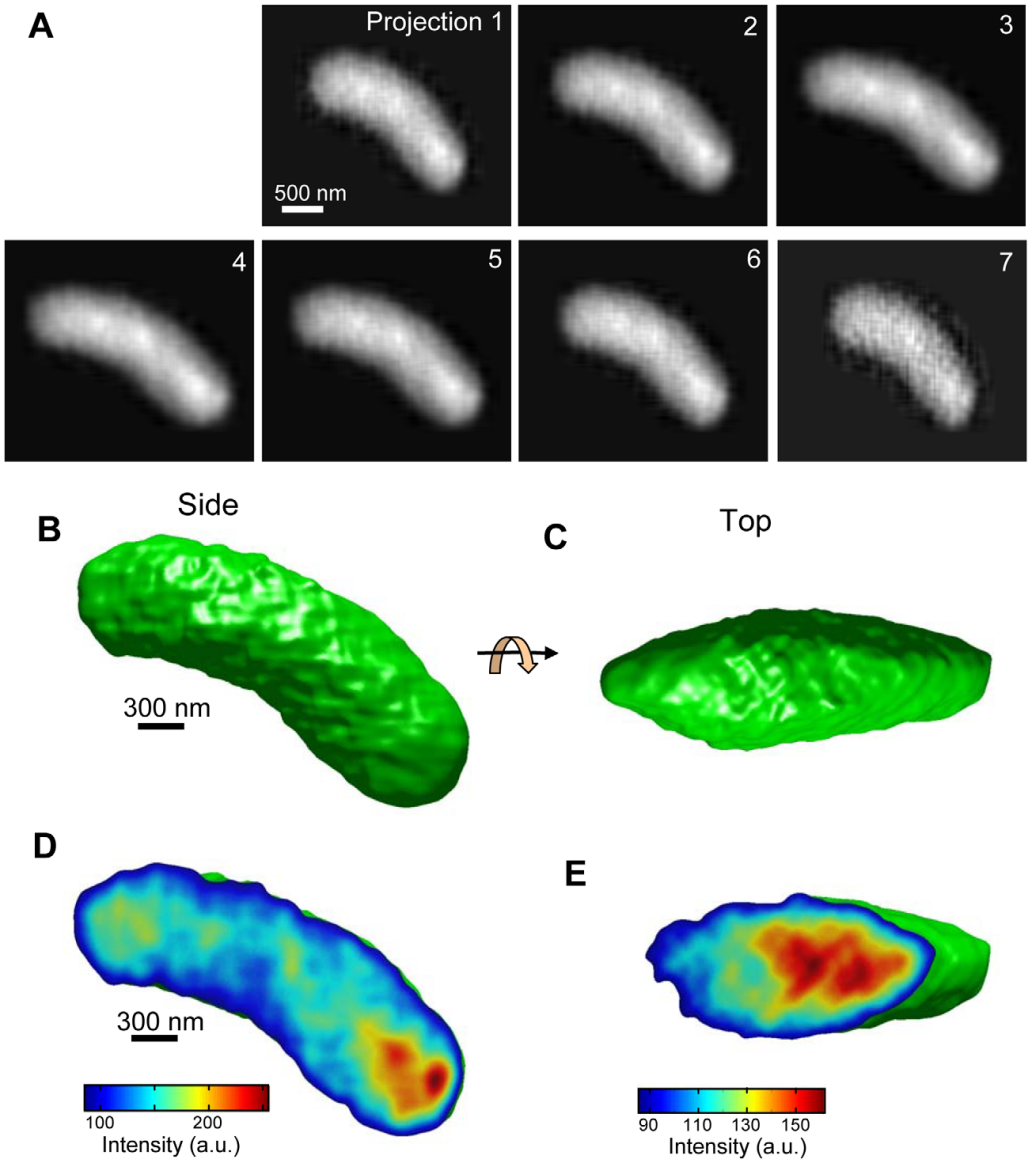
Final 3D model and its projections. (A) Each projection (1 to 7) from the final 3D volume corresponds to the views from the X-ray PDs (−40.2° to 45°). These images are very similar to the original X-ray images (Figure 2B). (B) Surface-rendered image of a bacterium is obtained by using 2σ that is greater than the mean value of the final 3D model. (C) Top view of 3D image of a bacterium, obtained by 90° rotation of 3D view shown in (B). (D) Cross section parallel to the vertical axis through the 3D volume with colour map to indicate density. The high-density region is coloured red and is visible at the right bottom end of the bacterium. (E) Cross section perpendicular to the vertical axis. The high-density region is located at the right side of the bacterium.

Figure 6 shows the projections and the 3D volume once annealing is complete. Each model projection shown in Figure 6A is very similar to the X-ray images for the corresponding tilt angle (Figure 2B). Therefore, the calculated 3D volume is likely to be highly reliable. The 3D volume viewed from the side and the top and the corresponding cross sections are shown in Figure 6B–E; Figure 6B and C show that the bacterium has the form of a slightly curved cylinder. A higher-density area in the bacterium, which is probably the nucleoid, is located at the bottom right, and the point with slightly above-average density is detected at the top left in the bacterium (Figure 6D and E). The bacterium centre exhibits a complex grain structure with relatively low density.

**Figure 7:**
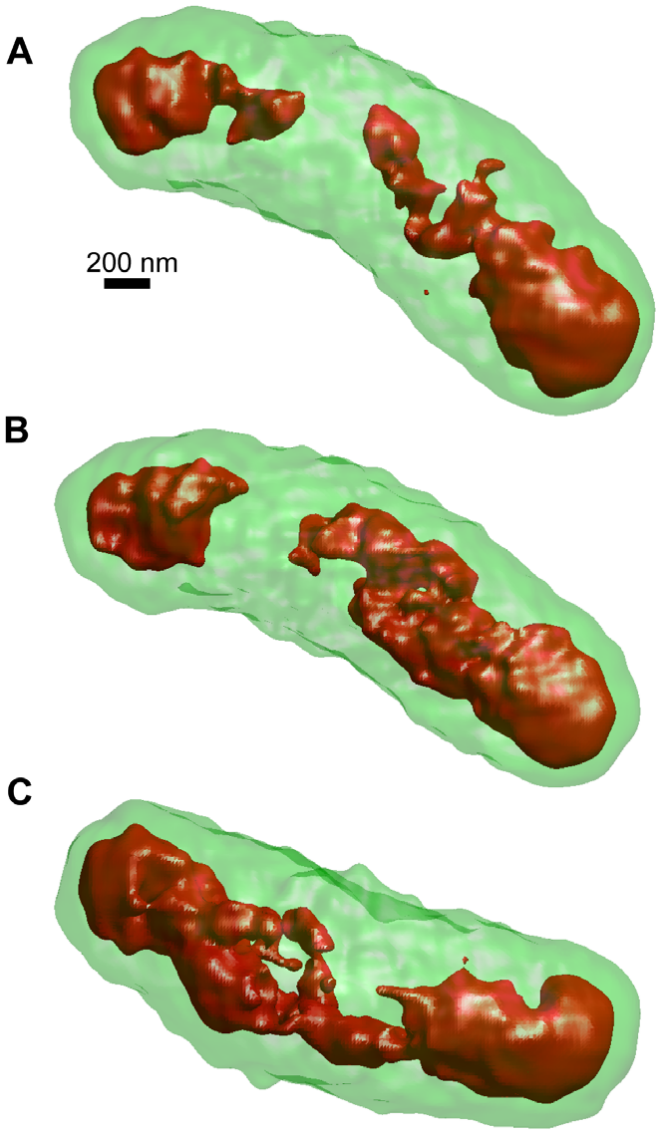
High-density areas in reconstructed 3D model of bacteria. (A) Red regions indicate >6.5σ above the average intensity. The high-density red regions are clearly located at both ends of the bacterium, which likely suggests the presence of a nucleoid. The complex structure was detected in the central area of the bacterium. (B), (C) High-density regions in the other two bacteria. Both structures indicated that high-density regions exist at the extremities of the bacteria.

To clearly identify the high-density region, we captured images of three bacteria of similar length and have displayed their inner structures in Figure 7. Figure 7A shows the same bacterium that is displayed in Figure 6 and reveals that the red high-density regions are clearly located in the longitudinal extremities of the bacterium. Furthermore, the complex inner structure of this bacterium is clearly observed. These features of the high-density regions shown in Figure 7A are similar to those observed in the other two bacteria shown in Figure 7B and C.

## Discussion

The results presented above demonstrate that the proposed system (Figures 1–4) can obtain a high-resolution 3D structure of the specimens in an atmospheric unstained sample (Figures 5–7). These images clearly reveal the complex structure of the high-density regions of the bacteria. The enabling technology is the linear array of X-ray PD elements that simultaneously capture a sequence of tilt X-ray images without requiring sample rotation or tilt (Figures 1 and 2). Therefore, the proposed system can calculate the 3D volume at the point of sample fixing, which enables high-speed 3D detection. In addition, this aspect of the technology further contributes to reducing sample damage.

The spatial resolution of the proposed system can be estimated from the geometry of the X-ray source diameter, the PD size and the separation between the two [18]. X-ray detector XPD-4 is positioned directly under the sample at a distance of 13 mm and presents a rectangular cross section of 2 mm×5 mm (in the *x* and *y* directions, respectively, Figure S4A). For a 5-kV EB acceleration voltage, a Monte Carlo simulation using CASINO version 2.42 [28] (Figure S4B and C) indicates an X-ray source diameter of approximately 25 nm. The area of the sample covered by the X-ray beam expands in proportion to the depth from the X-ray emission point in the CuZn-coated film (Figure S4A). The spatial resolution of the image captured by XPD-4 is 78 nm×160 nm for a bacterium centred (z = 300 nm depth) (Figure S4D). At the right side of the PD array, XPD-7 views the sample at 45° with respect to EB (Figure S4A) and is 18.4 mm from the sample. Its resolution is 52 nm×92 nm at a depth of 300 nm, which is superior to the resolution of XPD-4. However, the X-ray image generated by XPD-7 is noisy because it is farther from the sample than XPD-4. Accordingly, more sensitive X-ray detectors with smaller cross sections are required to improve the 3D resolution beyond what is reported here. Furthermore, because it has many X-ray detectors, the 2D PD array is a suitable geometry for generating the high-resolution 3D model.

In the future, the system should be able to produce real-time 3D models using highly sensitive 2D X-ray detectors. Furthermore, work is in progress to develop an easy-to-use water-sample holder. A combination of these systems is expected to enable high-resolution observation of real-time movements of biological samples in water.

In conclusion, we have developed an X-ray CT system called 3D-SGXM that enables the observation of the 3D structure of atmospheric unstained specimens. The specimens are placed under a CuZn-coated Si_3_N_4_ film and the ensemble is secured in an atmospheric sample holder, so that the samples are in an atmospheric environment. Tilt X-ray images of the sample are detected by a linear X-ray PD array, and the sample's 3D structure is generated by a newly developed 3D reconstruction method that uses an SA algorithm. The 3D reconstructions presented herein clearly reveal the inner structure of the bacterium samples. This method can be easily used for diverse samples in a broad range of scientific fields.

## Materials and Methods

### CuZn coating on Si_3_N_4_ film

A 50-nm-thick Si_3_N_4_ film supported by a window (0.5 mm×0.5 mm) in a Si frame (5 mm×5 mm and 0.2 mm thick; Silson Ltd., UK) was coated with brass (70% Cu, 30% Zn) using a magnetron sputter machine model MSP-30T (Vacuum Devices, Inc., Japan). The sputtering conditions were 1.1 Pa Ar, 150 mA and 30 s sputtering time. The distance between the sputter target and the Si_3_N_4_ film was 50 mm. The deposited CuZn layer was approximately 60 nm thick, as determined by the sputter conditions.

### Sample preparation and an atmospheric sample holder

The biological sample of *Rhodobacter capsulatus*, obtained from the Shimatec Co. (Japan, Osaka), is a purple, rod-shaped, nonsulfur photosynthetic bacterium with flagella [26], [27]. One millilitre of bacterium solution was centrifuged at 6200 rpm for 1 min in a Capsulefuge PMC-060 (Tomy, Inc., Japan), and the supernatant solution was replaced by a 1% (w/v) trehalose solution of 1 ml (Hayashibara, Inc., Japan). To prepare the SEM sample, 2 µl of the bacterium solution was dropped onto a CuZn-coated Si_3_N_4_ film. After 1 min, the solution on the film was removed by filter paper and the film was dried in atmosphere at room temperature (23°C) for 5 min. The sample thus attached to the Si_3_N_4_ film was secured in the atmospheric sample holder (Figure 1).

The new sample holder maintains the sample at atmospheric pressure in the sample space between a CuZn-coated 50-nm Si_3_N_4_ film and a 100-nm Si_3_N_4_ film (Figure 1A). The sample space between the two Si_3_N_4_ films is sealed by two sample-holding pieces and two O-rings, and the holding pieces are coupled by screws. The biological sample in the atmospheric sample holder is then mounted onto the sample stage above the linear PD array (Figure 1B and D).

### Scanning electron microscopy and X-ray imaging system

The stage containing the sample was transferred to the chamber of a thermionic emission SEM (JSM-6390, JEOL, Japan). X-ray images were captured by an original SE detector and the linear X-ray PD array under high-vacuum conditions with the following parameters: magnification = 4000–30,000×, image size = 1280×960 pixels, observation time = 160 s, working distance = 7 mm, EB accelerating voltages = 5 kV and EB aperture = 42–45. For the bacterium sample imaged at 4000 and 30,000× magnifications (Figure 2), the X-ray images from the SE detector were filtered by a 2D Gaussian filter (GF) (size = 9×9 pixels, σ = 1) by using Matlab R2007b (Math Works Inc., USA). The image contrast level was normalized to black (low intensity) and white (high intensity).

A linear X-ray PD array model AXUV-16EL (IRD Inc., USA) was fixed under the sample and consisted of seven PD elements out of the possible 16 elements (Figure 1). The XPD output was recorded using a data recorder (EZ7510, NF Co., Japan) after amplification by PD preamplifiers (gain = 100× MA/V) and the main amplifiers (gain 100×), which consist of AD8562 (Analog devices Inc., USA) integrated circuits. The X-ray and EB scan signals were recorded in a 16-bit data recorder with 20-kHz sampling rate. After the experiments, the data files were transferred to a personal computer (Intel Core2 Duo E6850, 3.0 GHz, Windows XP), and the X-ray images were generated from the XPD signals combined with the EB scan signal, with the calculation performed by Matlab R2007b.

### Correction of X-ray PD images and image processing

Each linear XPD element detects a tilt image according to its position. With respect to EB, the angles *θ_x_* between the EB-irradiated spot on the sample and the PD elements range from −40.2° to 45.0° (Figure S2C). The detection length of each XPD is shortening from the distance of EB scanning on Si_3_N_4_ film by its set angle of *θ_x_*, these reduction rates are calculated by cos(*θ_x_*) (Figure S2B). Therefore, horizontal ratio at each constructed X-ray image is expanded by the EB scan signal at its XPD setting position, because the observed length by scan signal is based on a detector just under EB point. To recover the true tilt images, the horizontal width of the detected images are reduced by the factor cos(*θ_x_*). Furthermore, to generate the 3D model, each X-ray signal must be transformed into X-ray absorbance (*A_n_*), which is done using Eq. (1):
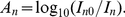
(1)In Eq. (1), *I_n_* is the X-ray intensity of each X-ray element *n* and *I_n_*_0_ is the base intensity without the sample.

After correction, the X-ray images were filtered by a 2D Gaussian filter (GF, size = 5×5 pixels, σ = 1) via Matlab R2007b. The images were normalized to 8-bit contrast. For 3D reconstruction from the X-ray images, the 1280×960 pixel images were reduced to 320×240 pixels.

### 3D reconstruction algorithm

To generate a 3D model from seven XPD images, we developed a highly accurate reconstruction method that uses an SA algorithm (Figures 3 and 4) that was implemented by a Matlab script. First, a 3D mask was calculated for each 2D mask from the X-ray images (Figure S3). This approach contributes to the short calculation time because it reduces the 3D space involved. The 3D voxel values and all projections were initially set to 0. Starting from the initial value of 0, a position in the 3D mask is randomly selected, and the voxel values in the kernel area at the selected position are randomly shifted. Initially, the kernel area is set to a 3D normal distribution with SD = 15σ, and the randomly changed value in the kernel is SD = 0.2σ, which is gradually reduced at each calculation cycle.

The new 3D volume is reprojected onto the seven directions corresponding to the seven angles of the XPD elements, and the error between each reprojection and its corresponding X-ray image is calculated by the residual sum of the squares. This calculated error is used by the SA algorithm to determine whether to accept the modified volume [29]. If the new error value is less than the previous error value, the modified volume is accepted unconditionally. If the error has increased, the Boltzmann probability factor of *P*(*ΔE*) is calculated using Eq. (2) and is used to determine whether to accept the modified voxel value.
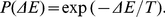
(2)In Eq. (2), *ΔE* is the change in the error and *T* is the current temperature. A random number (*R*) uniformly distributed in the interval 0 to 1 is generated and the volume change is accepted if *R*<*P*(*ΔE*). If *R*>*P*(*ΔE*), the volume is returned to its previous state.

Next, a new position is selected at random, its volume is randomly shifted and the SA algorithm is applied again. This procedure is iterated 100,000 times. When finished, the temperature, the kernel size and its value-shifting SD decrease exponentially. Furthermore, this entire cycle is repeated 200 times. The temperature, the kernel size and its shifting value are initially set to 10, 15σ and 0.2σ, respectively. After the algorithm is executed, these values are reduced to 0.1, 2σ and 0.1σ, respectively.

## Supporting Information

Figure S1 **Energy of X-rays emitted by the interaction of EB with CuZn-coated Si_3_N_4_ film.** (A) Overview of the apparatus to measure the X-ray photon energy, which uses an energy dispersive X-ray spectrometer (EDS) model EX-2100 including SEM model JSM-5601 (JEOL, Japan). EB irradiates the centre of the CuZn-coated Si_3_N_4_ film on the Al stage. The EDS detector is positioned 80 mm from the EB-irradiated spot and at a 30° angle with respect to EB; its detection area is 10 mm^2^. SEM is operated at 5–7 kV for the EB acceleration, 400× magnification and an EB aperture of 40. The X-ray-acquisition time is 100 s. (B) EDS spectrum for 5-kV EB acceleration. The large peak at 1 keV is due to both Cu and Zn X-ray lines. The weak peak at 1.8 keV is the Si X-ray line, and its presence suggests that a small number of electrons are transmitted to the Si_3_N_4_ film through the CuZn layer. However, under these conditions, the 1.5-keV Al peak is not detected (from the Al stage), which suggests that the impinging electrons do not cross the CuZn-coated Si_3_N_4_ film. (C) EDS spectrum for 7-kV EB acceleration. Under these conditions, a weak Al peak is detected from the Al stage that is positioned under the CuZn-coated Si_3_N_4_ film. Therefore, in this case, the 7-kV impinging electrons penetrate the CuZn-coated Si_3_N_4_ film.
(TIF)

Figure S2 **Schematic diagram of tilt imaging system based on the linear X-ray PD array.** (A) Overview of tilt imaging system showing left, right and centred PD elements of the linear X-ray PD array. A left-side PD element detects the left-tilt image of the sample, because the X-rays arriving at the detector are tilted to the left by the sample. Likewise, the right-side PD element detects the right-tilt images. (B) Schematic showing the influence of different detection angles on the detected length *Px_i_*. The left-side PD located at *θ*_1_ detects the EB-scanned width of *E_x_* reduced by cos(*θ*_1_), [*P_x_*_1_ = *E_x_*cos(*θ*_1_)]. The centre PD element (under the sample) detects the same width as the EB-scanned length, [*P_x_*_2_ = *E_x_*]. (C) Schematic showing the angles of each X-ray PD element. The linear X-ray PD array is positioned 13 mm under the sample. Each PD element is a 2×5 mm^2^ rectangle, and there are seven active signal-detection elements from among the 16 elements. The angles between the EB-irradiated spot and the PD elements are −40.2°,−28.1°,−13.0°, 4.4°, 21.0°, 34.7° and 45.0°.
(TIF)

Figure S3 **Outline of 3D mask calculation.** First, the 2D X-ray-image masks are calculated with 2σ larger than the image's average intensity. The intensity of this 2D mask is normalized to range from 0 to 100. Each 2D mask is back projected onto the angle corresponding to the PD. The 3D mask is obtained from the specific threshold of 600 for the 3D volume by the mask back-projections.
(TIF)

Figure S4 **Spatial resolution of 3D-SGXM.** (A) Overview of the sample detection area for two X-ray PD elements XPD-4 and XPD-7. The X-ray detection area expands gradually at deeper positions. XPD-4 is positioned 13 mm directly under the sample and measures 2 mm×5 mm. XPD-7 on the right side of the linear PD array is oriented at 45° with respect to EB and from the EB-irradiated spot. It is approximately 18.4 mm from the EB-irradiated position. (B) MC simulation showing electron trajectories in the 50-nm Si_3_N_4_ film coated by a 60-nm CuZn layer. A 20-nm EB spot diameter and a 5-kV accelerating voltage conditioned the simulation. (C) Normalized intensity of CuZn characteristic X-rays as a function of radial distance in the CuZn layer by MC simulation. The X-ray spot radius was 12.5 nm. (D) Estimated spatial resolution of XPD-4 and XPD-7. The spatial resolution of XPD-4 and XPD-7 consists of an *x* component (labelled *a*) and a *y* component (labelled *b*).
(TIF)
