# Supplementary figures and images for: Three-Dimensional X-ray Observation of Atmospheric Biological Samples by Linear-Array Scanning-Electron Generation X-ray Microscope System
Source: PLoS One. 2011 Jun 23;6(6):e21516. doi: 10.1371/journal.pone.0021516 (PMC3121776; doi:10.1371/journal.pone.0021516)

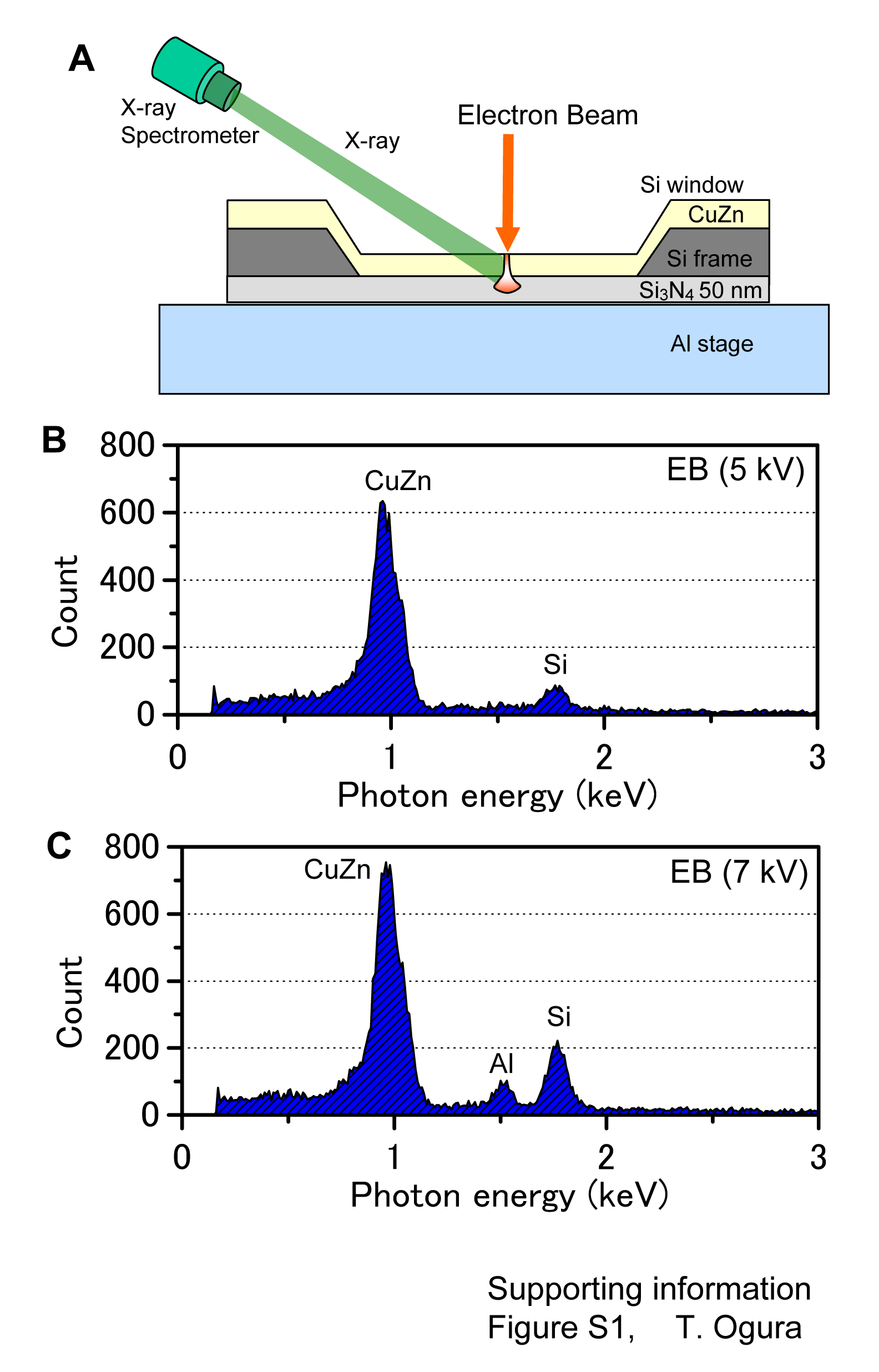

Supplement: Figure S1 — Energy of X-rays emitted by the interaction of EB with CuZn-coated Si3N4 film. (A) Overview of the apparatus to measure the X-ray photon energy, which uses an energy dispersive X-ray spectrometer (EDS) model EX-2100 including SEM model JSM-5601 (JEOL, Japan). EB irradiates the centre of the CuZn-coated Si3N4 film on the Al stage. The EDS detector is positioned 80 mm from the EB-irradiated spot and at a 30° angle with respect to EB; its detection area is 10 mm2. SEM is operated at 5–7 kV for the EB acceleration, 400× magnification and an EB aperture of 40. The X-ray-acquisition time is 100 s. (B) EDS spectrum for 5-kV EB acceleration. The large peak at 1 keV is due to both Cu and Zn X-ray lines. The weak peak at 1.8 keV is the Si X-ray line, and its presence suggests that a small number of electrons are transmitted to the Si3N4 film through the CuZn layer. However, under these conditions, the 1.5-keV Al peak is not detected (from the Al stage), which suggests that the impinging electrons do not cross the CuZn-coated Si3N4 film. (C) EDS spectrum for 7-kV EB acceleration. Under these conditions, a weak Al peak is detected from the Al stage that is positioned under the CuZn-coated Si3N4 film. Therefore, in this case, the 7-kV impinging electrons penetrate the CuZn-coated Si3N4 film. (TIF) [file pone.0021516.s001.tif]

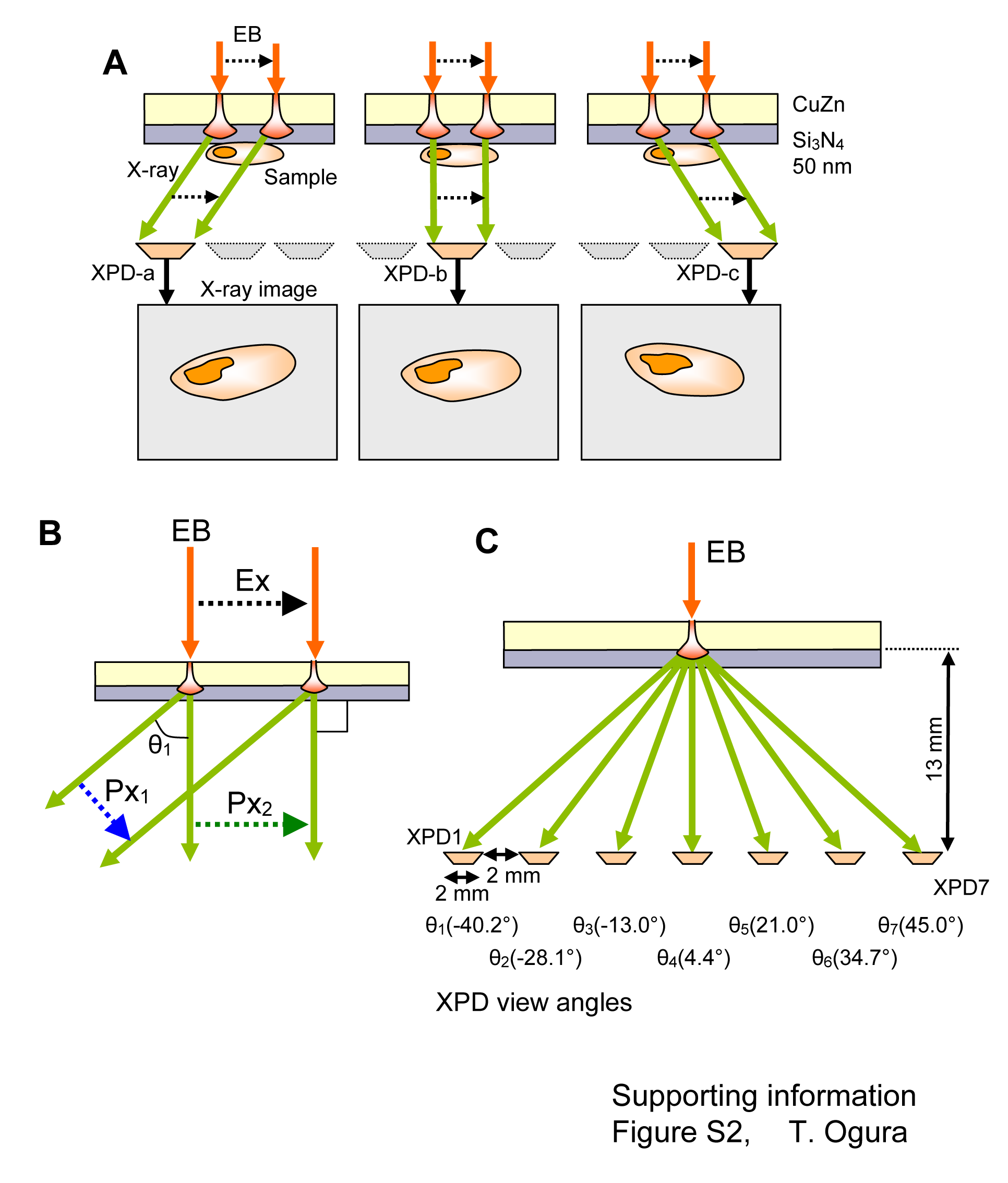

Supplement: Figure S2 — Schematic diagram of tilt imaging system based on the linear X-ray PD array. (A) Overview of tilt imaging system showing left, right and centred PD elements of the linear X-ray PD array. A left-side PD element detects the left-tilt image of the sample, because the X-rays arriving at the detector are tilted to the left by the sample. Likewise, the right-side PD element detects the right-tilt images. (B) Schematic showing the influence of different detection angles on the detected length Pxi. The left-side PD located at θ 1 detects the EB-scanned width of Ex reduced by cos(θ 1), [Px 1 = Excos(θ 1)]. The centre PD element (under the sample) detects the same width as the EB-scanned length, [Px 2 = Ex]. (C) Schematic showing the angles of each X-ray PD element. The linear X-ray PD array is positioned 13 mm under the sample. Each PD element is a 2×5 mm2 rectangle, and there are seven active signal-detection elements from among the 16 elements. The angles between the EB-irradiated spot and the PD elements are −40.2°,−28.1°,−13.0°, 4.4°, 21.0°, 34.7° and 45.0°. (TIF) [file pone.0021516.s002.tif]

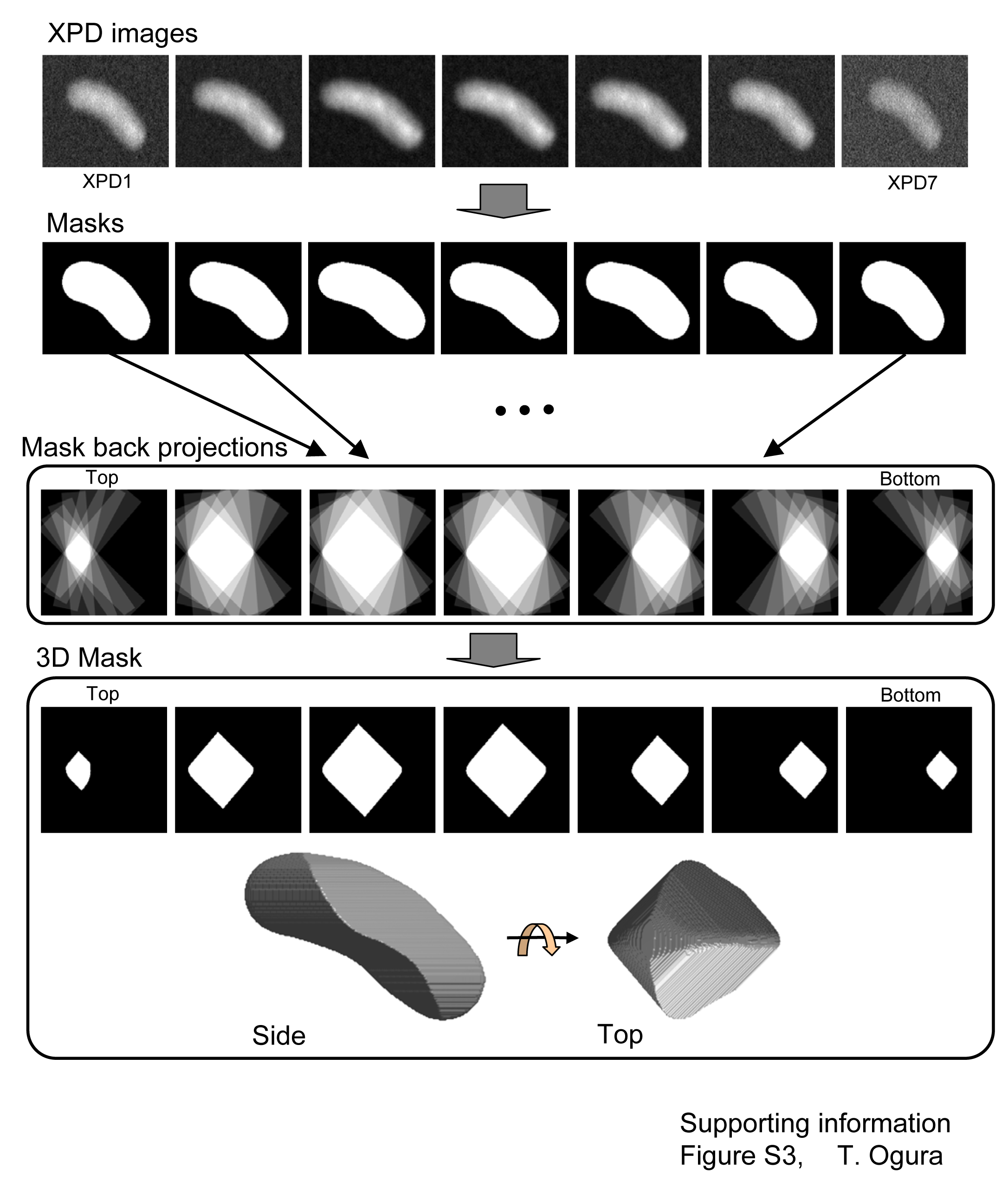

Supplement: Figure S3 — Outline of 3D mask calculation. First, the 2D X-ray-image masks are calculated with 2σ larger than the image's average intensity. The intensity of this 2D mask is normalized to range from 0 to 100. Each 2D mask is back projected onto the angle corresponding to the PD. The 3D mask is obtained from the specific threshold of 600 for the 3D volume by the mask back-projections. (TIF) [file pone.0021516.s003.tif]

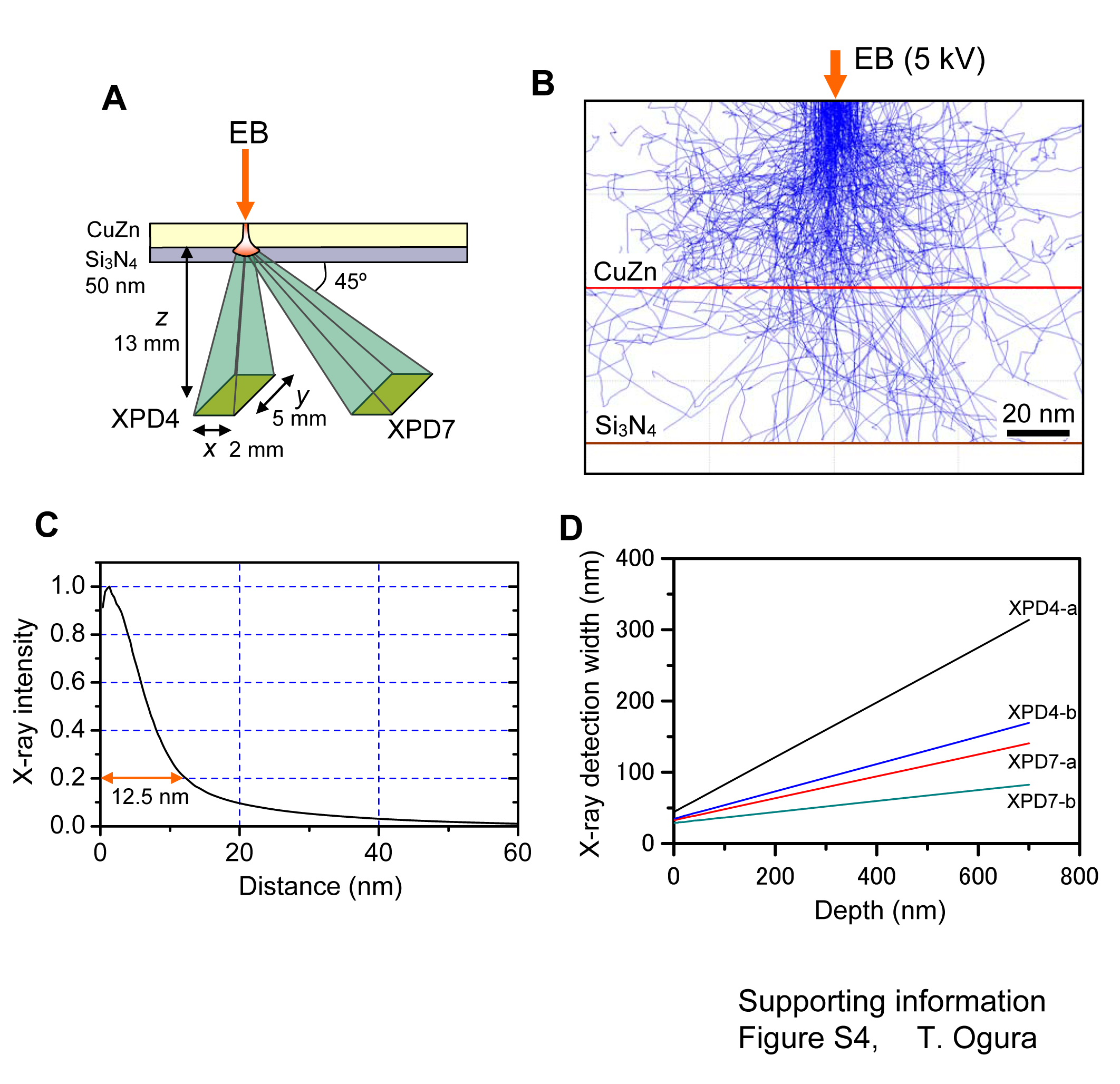

Supplement: Figure S4 — Spatial resolution of 3D-SGXM. (A) Overview of the sample detection area for two X-ray PD elements XPD-4 and XPD-7. The X-ray detection area expands gradually at deeper positions. XPD-4 is positioned 13 mm directly under the sample and measures 2 mm×5 mm. XPD-7 on the right side of the linear PD array is oriented at 45° with respect to EB and from the EB-irradiated spot. It is approximately 18.4 mm from the EB-irradiated position. (B) MC simulation showing electron trajectories in the 50-nm Si3N4 film coated by a 60-nm CuZn layer. A 20-nm EB spot diameter and a 5-kV accelerating voltage conditioned the simulation. (C) Normalized intensity of CuZn characteristic X-rays as a function of radial distance in the CuZn layer by MC simulation. The X-ray spot radius was 12.5 nm. (D) Estimated spatial resolution of XPD-4 and XPD-7. The spatial resolution of XPD-4 and XPD-7 consists of an x component (labelled a) and a y component (labelled b). (TIF) [file pone.0021516.s004.tif]
